# American Medical Association

**Published:** 1852-11

**Authors:** 


*American Medical Association.*

At a meeting of the Association, held at Richmond, Va., May, 1852, the undersigned were appointed a Committee to receive voluntary communications on medical subjects, and to award two prizes of one hundred dollars each, to the author of the best two essays.

Each communication must be accompanied by a sealed packet, containing the name of the author, which will be opened only in the case of the successful competitors. Unsuccessful communications will be returned on application, after the 1st of June, 1853.

Communications must be addressed (post paid) to the Chairman of the Committee, Dr. Joseph M. Smith, 56 Bleeker street, N. Y., on or before the 20th of March, 1853.

JOSEPH M. SMITH, M. D.,
JOHN A. SWETT, M. D.,
W. PARKER, M. D.,
GURDON BUCK, M. D.,
ALFRED C. POST, M. D.

New York, Sept. 17, 1852.

